# Evidence for a Supraspinal Contribution to the Human Crossed Reflex Response During Human Walking

**DOI:** 10.3389/fnhum.2018.00260

**Published:** 2018-06-29

**Authors:** Natalie Mrachacz-Kersting, Sabata Gervasio, Veronique Marchand-Pauvert

**Affiliations:** ^1^Department of Health Science and Technology, Aalborg University, Aalborg, Denmark; ^2^INSERM, CNRS, Laboratoire d’Imagerie Biomédicale (LIB), Sorbonne Universités, Paris, France

**Keywords:** crossed reflexes, cortical contribution, afferent feedback, human, walking

## Abstract

In humans, an ipsilateral tibial nerve (iTN) stimulation elicits short-latency-crossed-responses (SLCR) comprised of two bursts in the contralateral gastrocnemius lateralis (cGL) muscle. The average onset latency has been reported to be 57–69 ms with a duration of 30.4 ± 6.6 ms. The aim of this study was to elucidate if a transcortical pathway contributes to the SLCR. In Experiment 1 (*n* = 9), single pulse supra-threshold transcranial magnetic stimulation (supraTMS) was applied alone or in combination with iTN stimulation (85% of the maximum M-wave) while participants walked on a treadmill (delay between the SLCR and the motor evoked potentials (MEP) varied between −30 and 200 ms). In Experiment 2 (*n* = 6), single pulse sub-threshold TMS (subTMS) was performed and the interstimulus interval (ISI) varied between 0–30 ms. In Experiment 3, somatosensory evoked potentials (SEPs) were recorded during the iTN stimulation to quantify the latency of the resulting afferent volley at the cortical level. SLCRs and MEPs in cGL occurred at 63 ± 6 ms and 29 ± 2 ms, respectively. The mean SEP latency was 30 ± 3 ms. Thus, a transcortical pathway could contribute no earlier than 62–69 ms (SEP+MEP+central-processing-delay) after iTN stimulation. Combined iTN stimulation and supraTMS resulted in a significant MEP extra-facilitation when supraTMS was timed so that the MEP would coincide with the late component of the SLCR, while subTMS significantly depressed this component. This is the first study that demonstrates the existence of a strong cortical control on spinal pathways mediating the SLCR. This likely serves to enhance flexibility, ensuring that the appropriate output is produced in accord with the functional demand.

## Introduction

In animal studies, interneurons have been identified that receive input from sensory neurons arising from muscle receptors located on the ipsilateral side and target motoneurons innervating muscles located on the contralateral side (Jankowska, 2008). With current methodologies, it is not possible to directly test these commissural interneurons in the intact human. However, in a number of studies, several short-latency responses have been reported in contralateral muscles resulting directly from either an unexpected ipsilateral ankle or knee joint rotation or an ipsilateral electrical nerve stimulation (Berger et al., 1984; Dietz et al., 1986; Duysens et al., 1991; Stubbs and Mrachacz-Kersting, 2009; Stubbs et al., 2011; Gervasio et al., 2013; Stevenson et al., 2013; Hanna-Boutros et al., 2014). For example, a facilitation of the contralateral lateral gastrocnemius muscle (cGL) is evoked when the ipsilateral tibial nerve (iTN) is stimulated at an intensity producing an M-wave of 85% its maximal size (85% Mmax) (Gervasio et al., 2013). With the same stimulation, the ongoing electromyographic (EMG) activity of the contralateral soleus (cSOL) muscle is significantly depressed at a latency of 37–40 ms (Stubbs et al., 2011). The size of these responses is significantly modulated during walking, showing the largest effects during phase transitions. This corroborates findings in the lamprey where 60% commissural interneurons were found to be active during the transition phase with only 40% during the ipsilateral locomotor burst phase (Biró et al., 2008).

The onset latency of the short-latency response in the cSOL is too early for any inputs from supraspinal structures and the central processing delay for the cSOL inhibition has been confirmed to be approximately 3 ms (Hanna-Boutros et al., 2014). Selectively blocking specific muscle afferents eliminates or significantly modifies the amplitude of the response elicited in the contralateral muscles (Stubbs and Mrachacz-Kersting, 2009; Hanna-Boutros et al., 2014). It is thus likely that the mediator of these crossed responses, similar to animal studies, are commissural interneurons.

The short duration of the depression elicited in the cSOL following iTN stimulation suggests a purely spinal pathway. However, the facilitation elicited in cGL starts on average 57–69 ms following the stimulation and has a longer duration (Gervasio et al., 2013, 2015). Moreover, this response is characterized by several peaks (Gervasio et al., 2013). We speculated that the cGL response is likely mediated by supraspinal centers. A transcortical pathway would indicate a higher cortical control probably in order to ensure greater flexibility for the generation of an appropriate output in accord with the functional demand. We previously suggested that the facilitation observed in cGL may have the purpose of accelerating the propulsion phase of the contralateral leg, preparing, in this way, for a faster step in the event that the ipsilateral leg, which was stimulated, is unable to support the body weight. It is therefore likely that crossed responses would increase dynamic stability during walking (Gervasio et al., 2015).

The aim of the current study was to elucidate if a transcortical pathway contributes to the cGL short-latency-crossed-responses (SLCR) during human walking. We applied transcranial magnetic stimulation (TMS) using either a supra-threshold (supraTMS) or a sub-threshold stimulus (subTMS) for evoking a motor evoked potential (MEP) in the cGL. We hypothesized that the combination of iTN stimulation and supraTMS would elicit a more prominent response than the sum of the responses obtained when a single stimulation is performed, and that the subTMS would suppress the cGL response; such a result would prove the convergence between stimulation of ipsilateral afferents via iTN stimulation and activation of the corticospinal cells by TMS. Part of the results have been published in abstract form (Mrachacz-Kersting et al., 2014).

## Materials and Methods

### Participants

Fifteen right leg dominant participants (11 males, 4 females; age: 20–29 years) with no prior history of neurological conditions provided written informed consent prior to participating in one of two experiments. Nine participants partook in Experiment 1, six in Experiments 2 and 5 in Experiment 3. This study was carried out in accordance with the recommendations of the Scientific Ethics Committee of Northern Jutland. The protocol was approved by the Scientific Ethics Committee of Northern Jutland (Reference number: VN-20110040). All subjects gave written informed consent in accordance with the Declaration of Helsinki.

### Apparatus and Instrumentation

The muscle activity of the ipsilateral soleus (iSOL) and the cGL was recorded using disposable surface electrodes (20 mm Blue Sensor Ag/AgCl, AMBU A/S, Denmark). The EMG signals were amplified and band pass filtered between 20 Hz and 1 kHz. A pressure-sensitive trigger was placed under the heel of the participant’s ipsilateral leg. This was used to trigger the data collection and the stimulation. Data were sampled at 2 kHz.

Single pulses (with a posterior to anterior directed current) of non-invasive transcranial magnetic stimulation (TMS) were applied using a Magstim 200 (Magstim Company, Dyfed, UK) with a focal figure of eight double cone coil (110 mm diameter) to elicit a MEP in the cGL EMG.

An isolated stimulator (Noxitest IES 230) was used to apply monopolar stimuli to the right iTN. The cathode (PALs platinum round electrode, Model No. 879100, 3.2 cm diameter, Axelgaard Man) was positioned in the popliteal fossa and the anode (PALs platinum rectangular electrode, Model No. 895340, 7.5–10 cm, Axelgaard Man) on the anterior aspect of the knee, at the level of the patella. Initially, single stimuli were delivered every 3–5 s to locate a spot for the cathode where the least current was required to elicit an M-wave in the iSOL EMG.

### Experimental Procedures

Participants walked on a treadmill (Split 70/157/ASK; Woodway, Weil am Rhein, Germany) and, after an adaptation period of 2–3 min, were asked to select a preferred walking speed they feel comfortable with when having to walk for 30 min or more. Typically, a minimum of 5–10 min was provided to allow the participants to adapt to this walking speed and data acquisition is commenced. Stride time was monitored online to ensure minimal stride variability. Initially, data for 20 walking cycles was collected and the average stride time for these cycles calculated. During data collection, the crossed responses were elicited at 80% of the gait cycle expressed in relation to the stride time of the stimulated (ipsilateral) leg (Gervasio et al., 2013). In this phase, the ispilateral leg is in the swing phase while the contralateral leg is in mid-stance. The crossed response in the cGL has indeed been shown to be most prominent when elicited at this point of the gait cycle.

#### Peripheral Nerve Stimulation

**Figure 1:**
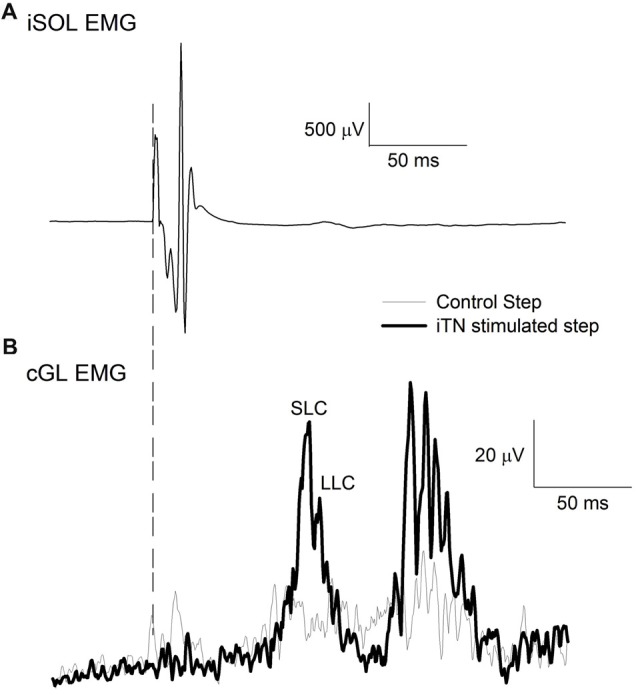
Sample traces of the ipsilateral soleus (iSOL) and the contralateral gastrocnemius lateralis (cGL). **(A)** The mean iSOL electromyographic (EMG) during ipsilateral tibial nerve (iTN) stimulation. **(B)** The mean rectified EMG of cGL during control and iTN stimulated trials. The early and late components are labeled short-latency component (SLC) and long latency component (LLC). All data are the average of 15 trials from *n* = 1.

For each participant, the iTN was initially stimulated while standing. The intensity of stimulation started at 5 mA and was increased in steps of 5 mA every three stimuli until the resulting M-wave no longer increased in its peak to peak size. This intensity served as the maximum intensity level for the subsequent walking experiment. Next, participants were asked to walk at their self-selected pace (0.97–1.11 m.s^−1^) and electrical stimulation was delivered to the iTN at 80% the gait cycle with randomized intensities. This ensured that the entire input-output relation of the stimulation intensity and the resulting M-wave was established. To ascertain stability of the peripheral nerve stimulation during locomotion, the resulting M-wave in the iSOL EMG was monitored online. The maximum M-wave (M-max) was extracted from the input-output curve for each participant and the intensity that produced an M-wave amplitude equal to of 85% M-max was determined. Data was then acquired with the participants walking while their iTN was stimulated at the selected time and intensity (Figure 1A). A control condition in which no stimulation occurred was also collected and stimulation and control trials were randomized. The onset of the facilitatory response in the cGL EMG following the iTN stimuli was extracted from the averaged EMG signal and used to establish the timing of the iTN and TMS stimuli for Experiment 1 and two outlined below. A typical trace of the average cGL activity during the control (thin trace) and stimulated (thick trace) conditions are shown in Figure 1B.

#### Transcranial Magnetic Stimulation

To find the optimal site for evoking a MEP in the cGL EMG, participants were seated in a chair and the TMS intensity set to 50% the maximal stimulator output (MSO). Commencing at the vertex, three successive stimuli were applied and the peak to peak amplitude of the cGL monitored online. Next, the position of the coil was varied randomly along a square grid with 1 cm distances between successive spots. Three stimuli were applied at each location (maximally 4–6 positions were thus tested per participant) and the peak to peak cGL MEP monitored. The optimal place for stimulation, also referred to as the hot-spot, was selected as that coordinate where the peak-to-peak amplitudes of the cGL MEP were greater than amplitudes of adjacent coordinates for the same stimulus intensity. For all participants, this site was located approximately 1–2 cm anterior to the vertex. This site was marked using a felt pen to ensure that the coil position was maintained throughout the experiments. A custom made brace (see Schubert et al., 1997) was used to fixate the coil on the hot spot and ensure that the stimuli were applied over the same area of the motor cortex during the following dynamic task.

### Experiment 1: Combined SupraTMS and iTN Stimulation

To test if the combination of iTN stimulation and supraTMS would elicit a more prominent response than the sum of the responses obtained when a single stimulation is performed, we used the spatial facilitation technique first introduced by Eccles and Lundberg (1957). Nine participants (three females; aged 23–25 years) took part in Experiment 1. They were asked to walk on the treadmill at their self-selected pace while TMS was applied at 46%–57% MSO depending on the participant. The amplitude of the resulting MEP was monitored online and the stimulation intensity was adjusted to produce as much as possible, a MEP with similar amplitude of the SLCR in cGL EMG. In the subsequent part of the experiment the intensity was maintained at this setting. Assuming that the motor unit recruitment might be similar for the two pathways (Nielsen et al., 1999), we ensured that the MEP and the SLCR were of similar size, suggesting that the two pathways have likely activated similar motor units. Attempts were made to maintain both responses reasonably low in size to avoid saturation of the motor unit pool. Subsequently participants were exposed to 14 conditions while maintaining walking at the self-selected pace. Ten of these involved a single TMS (supraTMS) delivered and timed for each participant in relation to the iTN stimulation to produce different inter-response intervals (IRI) so that the onset of the MEP occurred before (−30 and −15 ms), at the same time (0 ms), or after (+5, +10, +15, +20, +30, +45 and +200 ms) the onset of the cGL SLCR. IRIs were varied randomly. In addition, a control condition in which no stimuli occurred as well as one in which only iTN stimuli were applied and one in which only supraTMS was applied, were interspersed with the combined stimuli. The time between consecutive trials was set to 5–7 s. A total of 15 trials were collected for each condition.

### Experiment 2: Combined SubTMS and iTN Stimulation

SubTMS has been shown in previous studies to suppress the EMG activity of the target muscle with an onset slightly later (about 10 ms) than that of the MEP (Davey et al., 1994; Petersen et al., 2001). This suppression is due to the activation of short intracortical inhibition and is used to evaluate the cortical contribution to the EMG activity of the target muscle. The sub-threshold stimulus would therefore be expected to reduce the amplitude of the cGL in order to confirm our hypothesis that a cortical contribution to the crossed response in the cGL exists. Six participants (2 females; aged 20–29 years) partook in Experiment 2. As for the previous experiment, participants were asked to walk on the treadmill. The active motor threshold (AMT—defined as the highest stimulation intensity that elicits 5 of 10 consecutive MEPs of −200 μV) for evoking a MEP in the cGL was determined during walking and at 80% of the gait cycle. Next, participants were exposed to eight randomized conditions. In five conditions, a single TMS pulse was delivered at 90% AMT of active motor threshold (subTMS) timed in relation to the iTN stimulation at different intervals. These were individualized for each participant based on the IRI, so that the onset of the suppression elicited by subTMS occurred at 0 ms, or after (+5, +10, +15, +20, +30, +45 and +200 ms) the onset of the cGL SLCR. The three remaining conditions were performed to control the background activity (no stimulus) and the effects of isolated subTMS and iTN stimuli. A total of 40 trials per condition were collected with 5–7 s time between consecutive trials.

### Experiment 3: Somatosensory Evoked Potentials (SEPs) Following iTN Stimulation

The cortical potentials evoked by iTN stimulations were recorded over the sensory cortex with surface electrodes in five participants (3 males, 2 females, aged 20–27 years). The stimulation intensity was set to 1× motor threshold, the pulse width to 200 μs and the inter-stimulus interval was randomized between 200 ms and 220 ms according to the guidelines of the International Federation of Clinical Neurophysiology (Mauguière et al., 1999). The Somatosensory evoked potentials (SEPs) were band pass filtered between 0.05–500 Hz at a sampling rate of 2 kHz and a gain of 10,000 (bilateral ears-referenced). A minimum of 3000 traces were recorded while the participants were seated and ensemble averaged online. The onset of the SEP was defined as the first major deflection in the ensemble averaged record, as determined by visual inspection.

### Data Analysis

Figure 1B displays a typical response in the cGL EMG following iTN stimulation for one participant. For each individual participant data was averaged across conditions. The responses were quantified from the rectified and averaged EMG. The onset of the cGL response was defined as that time where the averaged cGL EMG of the stimulated gait cycle exceeded the value of the averaged cGL EMG of the control gait cycle for an amount of 2× the standard deviation in a time window of 25–120 ms after the stimulation (Gervasio et al., 2013). The response size in the averaged and rectified cGL EMG was quantified as the root mean square (RMS) in a window from MEP onset to MEP offset (Experiment 1) or from the onset to the offset of the subTMS-induced EMG suppression (Experiment 2). In the latter case, the suppression onset and offset were determined as in Petersen et al. (2001) using visual inspection. When iTN was delivered alone, the modulation of EMG activity was evaluated in the same windows of analysis. The algebraic sum of the effects of isolated iTN stimuli and isolated TMS was calculated from the cGL EMG RMS for each participant after subtracting the background EMG level for each condition within the same windows of analysis. This was compared to the response elicited by the combination of iTN and TMS at the different IRIs, to which the background level was also subtracted.

### Statistical Procedures

The data were tested for normal distribution using Q-Q plots. Mauchly’s test of sphericity was used to test the assumption of sphericity for repeated measures ANOVA. If correction for sphericity was needed Greenhouse-Geisser adjusted significant values were used. A repeated measured ANOVA was used to compare the magnitude of the MEP and of the short-latency and long-latency components (LLC) of the crossed response. A one-tailed paired sample Student’s *t* tests revealed if the combined iTN and TMS conditions evoked responses greater than the algebraic sum of the responses evoked by iTN and TMS delivered separately (Petersen et al., 1998b) for Experiment 1. The same test was used for the data recorded in Experiment 2, to evaluate whether the combined iTN and subTMS conditions evoked responses smaller than the algebraic sum of the responses evoked by iTN and subTMS delivered separately. The Holm-Bonferroni method was used to correct for multiple comparisons. For all experiments, statistical significance was set to *P* < 0.05.

## Results

### The Short-Latency-Crossed-Response

**Figure 2:**
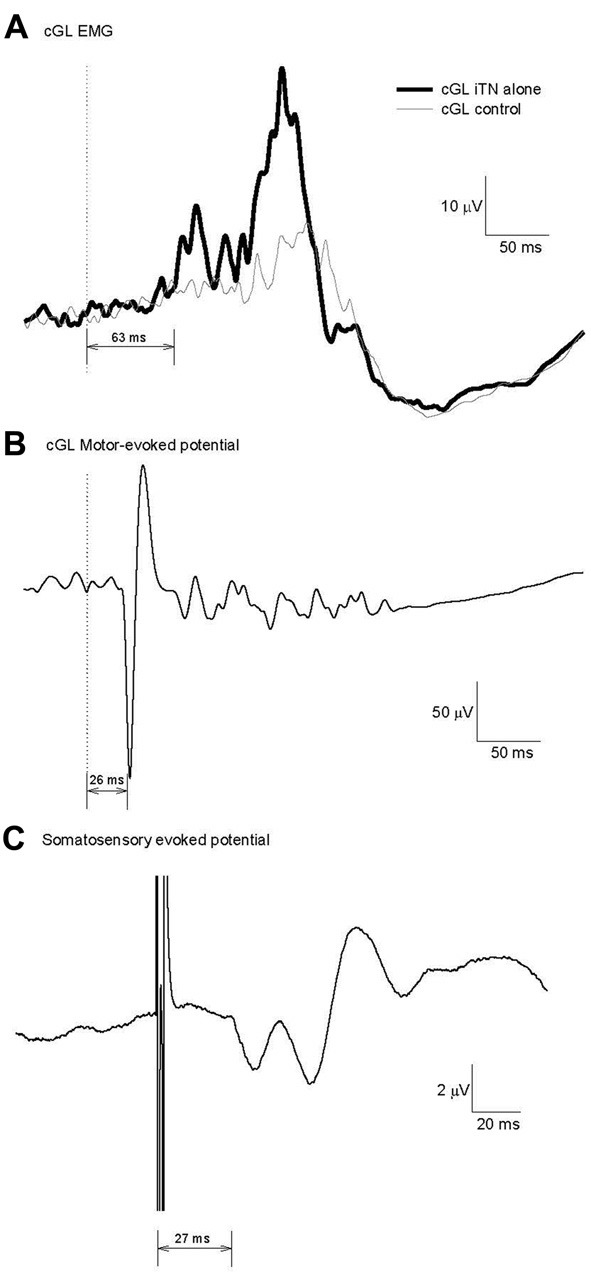
Calculation of the minimum conduction time necessary for a transcortical contribution to the cGL short-latency-crossed-responses (SLCR). **(A)** Reflex response evoked in the cGL by an imposed iTN stimulation at 80% of the gait cycle for one participant. Fifteen traces were averaged. **(B)** Motor evoked potential (MEP) in the cGL evoked by magnetic stimulation of the motor cortex. The latency is 26 ms. **(C)** Somatosensory evoked potential evoked after iTN as in **(A)**. The latency is 27 ms.

Figure 2A illustrates an example of an ensemble average data record (*n* = 15) for the cGL from a single participant when the iTN stimulation was applied alone at 80% of the gait cycle. The vertical dashed line indicates the time of the stimulus. In this example, the cGL responded with several bursts of activity commencing at 63 ms. Across all participants, the onset of the EMG facilitation in the cGL was 63 ± 6 ms and the duration 59 ± 27 ms. In all participants, at least two distinct peaks were observed, referred to as short-latency component (SLC) and LLC, respectively. The peak of the SLC occurred on average at 73 ± 7 ms and the LLC peak at 88 ± 6 ms. Similar to the ipsilateral stretch reflex, the background EMG activity does not always return to baseline levels prior to the LLC which is why no attempt was made to define its onset.

### Motor and Somatosensory Evoked Potentials

The fastest possible conduction time in the corticospinal efferent pathway was determined with the MEP elicited in the cGL EMG after TMS over the cortical primary motor area. Figure 2B shows the cGL MEP in an ensemble averaged data record for the same participant as in Figure 2A. The onset of the MEP was 26 ms and reflects the efferent conduction time for this participant (central + peripheral conduction time). SEPs produced by the iTN stimulation were recorded in *Experiment 3*, to estimate the fastest possible conduction velocity in the afferent sensory pathway to the cortex. The data presented in Figure 2C is the averaged SEP (*n* = 3000) for the same participant as in Figures 2A,B. The SEP latency was 27 ms. Thus, the earliest time at which a transcortical pathway could contribute to the cGL SLC in this participant was between 56 ms (26 + 27 + 3) and 63 ms (26 + 27 + 10) = MEP latency (efferent conduction time, 26 ms) + SEP latency (afferent conduction time, 27 ms) + the estimated central processing delay (3–10 ms) (Nielsen et al., 1997; Petersen et al., 1998b; Kurusu and Kitamura, 1999).

The mean MEP and SEP latencies across all participants were 29 ± 2 ms and 30 ± 3 ms, respectively. Therefore, a transcortical pathway has the potential to contribute to the cGL SLC no earlier than 57 ms (= (min MEP = 29 − 2) + (min SEP = 30 − 3) + (min central processing = 3)) following the iTN stimulation.

### MEP Facilitation as a Response to iTN

**Figure 3:**
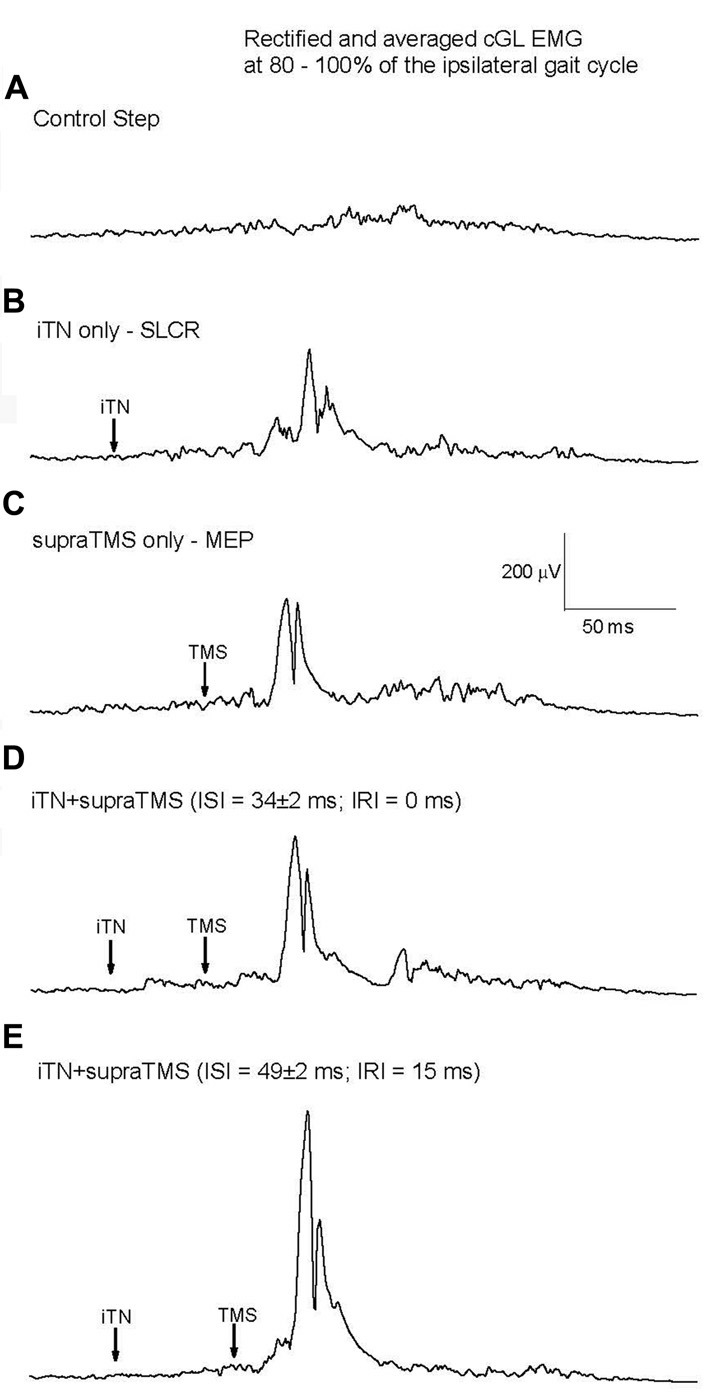
Responses elicited by iTN stimulation and transcranial magnetic stimulation (TMS) for one representative participant. **(A)** Mean rectified cGL EMG for the control (no stimulation) condition, **(B)** isolated iTN stimulation, **(C)** isolated TMS, **(D)** a combination of iTN and TMS timed so that the MEP’s and SLCR’s onset occurred at the same time and **(E)**: a combination of iTN and TMS timed so that the MEP commenced 15 ms after the SLCR’s onset.

TMS was applied so that MEPs occurred before (IRI −30 and −15 ms), at the same time (IRI 0 ms), or after (IRI: +5, +10, +15, +20, +30, +45 and +200 ms) the onset of the SLCR elicited by iTN stimulation at 80% the ipsilateral gait cycle, and the changes in MEP size in cGL were quantified. Considering that the SLCR has an average onset latency of 63 ms and the efferent conduction time is on average 29 ms (as assessed by the MEP onset latency), TMS has to be delivered at an average interstimulus interval (ISI) of 34 ms after iTN for a simultaneous arrival (central delay of 0 ms) at the motoneuron level of the cGL. Figure 3 shows the cGL EMG (average of 15 trials) from one representative participant without stimulation (background activity; Figure 3A), after isolated iTN stimulation (Figure 3B, onset latency of the cGL SLCR = 70 ms), after isolated TMS (Figure 3C), after combined iTN stimulation and TMS applied so that the onset of the MEP coincided with the onset of the SLC in the cGL EMG (ISI = 41 ms, corresponding to an IRI 0 ms; Figure 3D), and with a 15-ms longer interval between iTN and TMS (ISI = 54 ms) so that the onset of the MEP occurred 15 ms after the onset of the SLCR (IRI: + 15 ms; Figure 3E). The last condition has the purpose of studying the interaction between the MEP and the LLC; which has an average onset of 15.1 ± 3.7 ms after the onset of the SLC. No significant difference was shown between the size of the MEP and the size of the SLC and of the LLC of the crossed response when elicited separately (*F*_(1,09)_ = 2.95, *P* = 0.125).

**Figure 4:**
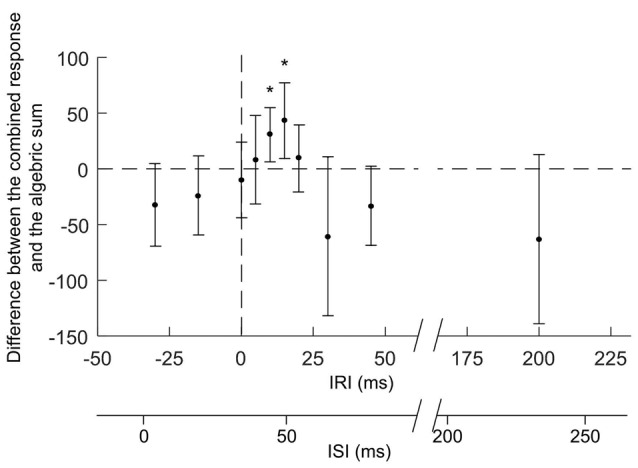
Responses elicited by the combination of iTN and TMS with different inter-response intervals (IRI). The figure reports mean values (and SD) of the magnitude of the combined responses from which the algebraic sum of the SLCR and MEP elicited separately for the respective IRI have been subtracted. The horizontal dashed line indicates no differences between the combined response and the algebraic sum of the two responses elicited separately. MEPs were elicited by TMS delivered at different timings (IRIs: −30, −15, 0, +5, +10, +15, +30, +45 and +200 ms) relative to the onset (time 0) of the SLC of the cGL SLCR. The second x-axis represents the interstimulus intervals (ISIs) between the iTN and the TMS stimulus (ISIs: 4 ± 2, 19 ± 2, 34 ± 2, 39 ± 2, 44 ± 2, 49 ± 2, 54 ± 2, 64 ± 2, 79 ± 2, 95 ± 2 and 234 ± 2 ms). The asterisks indicate significant difference between conditions.

The effects on combined stimuli related to the algebraic sum of SLC + MEP (indicated by the 100% dotted line in Figure 4) were compared at different IRIs (Figures 4–also shown are the ISIs). When MEPs were elicited either 10 ms (IRI +10 ms, *t*_(7)_ = −3.603, *P* = 0.0045) or 15 ms after SLCR (+15 ms, *t*_(6)_= −3.590, *P* = 0.0045) the combined stimuli produced an extra facilitation in cGL EMG, which was significantly larger than the algebraic sum of the SLCR + MEP. For these IRIs, the ISIs between iTN and TMS was 44 ± 2 ms and 49 ± 2 ms, respectively. In all other IRIs, the effects on combined stimuli were not different from the algebraic sum of the MEP + SLCR. Further, a rmANOVA with the factor “IRI” revealed no significant differences in the level of the cGL background muscle activation (*F*_(11,77)_= 2.04, *p* = 0.172).

### Changes in the Depression Following SubTMS as a Response to iTN

**Figure 5:**
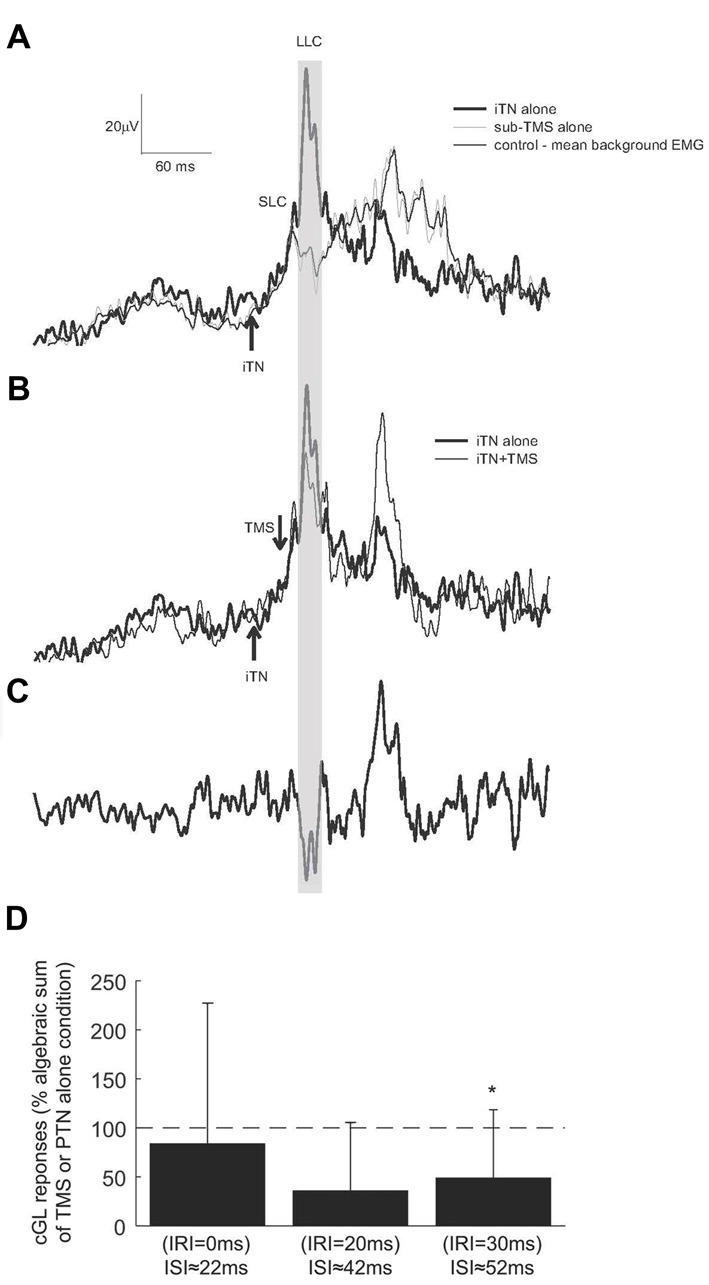
Responses elicited by iTN stimulation and subTMS. **(A)** The averaged rectified EMG of the cGL for the control (thin trace), isolated iTN stimulation (thick trace) and isolated TMS alone (gray trace) conditions is shown for one representative participant. **(B)** The cGL EMG is shown for the isolated iTN stimulation condition (thick trace) and the iTN+TMS at an IRI of 20 ms (ISI: 59 ± 2 ms; thin trace) condition. **(C)** The iTN+TMS at an IRI of 20 ms is subtracted from the isolated iTN condition (the two traced in **B**). The time window in which the depression occurs is evidenced by the gray shaded area. **(D)** The magnitudes of responses elicited when iTN and subthreshold TMS were applied to evoke IRIs of 0, 20 and 30 ms (ISIs: 29 ± 2, 49 ± 2 and 59 ± 2 ms) are here shown for all participants. Results are expressed as the algebraic sum of the responses elicited with TMS alone and iTN stimulation alone. The dashed horizontal line indicates 100% (no difference). The asterisks indicate significant differences (*p* < 0.05).

Figure 5 shows the cGL EMG from one participant following the iTN alone condition (thick trace, Figure 5A). For this participant facilitation commenced 63 ms following stimulation. When subTMS was applied alone, (TMS intensity set to 32% MSO corresponding to 90% of active motor threshold, the suppression of the cGL EMG occurred at 41 ms with a duration of 10 ms (gray trace in Figure 5A). Data in Figure 5B shows the effect of combined iTN stimulation and subTMS. The two stimuli were timed so that the suppression elicited by subTMS occurred 20 ms after the onset of the SLC (IRI +20 ms), i.e., +5 ms after the LLC, corresponding to the TMS pulse being delivered 42 ms after the iTN stimulus. The large suppression at the time of the late peak is visualized in Figure 5C where the combined condition is subtracted from the iTN only condition.

Across all participants, the mean latency of the inhibition was 41 ± 7 ms. A substantial depression of the SLCR was quantified only when the subTMS suppression was timed to occur at 20 and 30 ms after the onset of the crossed response (Figure 5D), corresponding to the convergence of the subTMS pulse with the LLC. At these times, the TMS was delivered respectively around 42 and 52 ms following the iTN stimulus. When the SLCR and the subTMS suppression were elicited with an IRI 30 ms (ISI 52 ms), the observed suppression was significantly different (*t*_(5)_= −3.211, *P* = 0.012) than the algebraic sum of the responses elicited separately. No significant differences were observed at other IRIs. In addition, a rmANOVA with the factor “IRI” revealed no significant differences in the level of cGL background activation (*F*_(5,25)_ = 3.379, *p* = 0.08, Partial Eta squared = 0.403).

## Discussion

In this study, we investigated if a transcortical pathway contributes to the late component of the cGL crossed response during human walking. In Experiment 1, we showed a significant facilitation of the response when TMS was combined with iTN but only when the induced MEP was timed to arrive with the LLC of the crossed response. In Experiment 2, we provided further evidence of the transcortical nature by showing a considerable depression but only when the subTMS was timed so that it elicited a suppression that coincided with the LLC.

TMS was timed for each participant individually such that the MEP arrival would correspond with the time of various components of the crossed reflex. If we consider the average SEP latency of 30 ± 3 ms, a central processing time of 3–10 ms and a MEP latency of 29 ± 2 ms, then no extra facilitation due to a cortical contribution may be expected if the TMS pulse is delivered 33–43 ms after the iTN stimulus. Indeed, for Experiment 1, we report a significant effect when the difference between the two stimuli (ISI) was on average 44 ± 2 and 49 ± 2 ms and for Experiment 2 on average 49 ± 5 and 59 ± 5 ms.

There has been increasing evidence to suggest that interlimb coordination during walking is largely controlled via commissural interneurons in various animal preparations (Butt et al., 2002a,b; Butt and Kiehn, 2003; Jankowska, 2008). These receive inputs from group II afferents (some di- or tri-synaptic input from reticulospinal neurons), reticulospinal neurons, group I afferents, lateral vestibular nucleus and/or reticulospinal tract, as well as those located in lamina VI–VII with input from group I and II afferents and the reticulospinal tract (Jankowska et al., 2005, 2009). In humans, it is difficult to investigate these interneurons directly. However, a number of studies have provided evidence for crossed reflexes following either peripheral nerve stimulation or ankle or knee joint perturbations (Berger et al., 1984; Dietz et al., 1986; Duysens et al., 1991; Stubbs and Mrachacz-Kersting, 2009; Stubbs et al., 2011, 2012; Gervasio et al., 2013, 2017; Stevenson et al., 2013; Hanna-Boutros et al., 2014; Mrachacz-Kersting et al., 2017). These responses are not to be compared to those termed the crossed extensor reflex. First, they occur at a significantly shorter latency (37–41 ms for cSOL, 57–69 ms for cGL following nerve stimulation; 62 ms for the contralateral biceps femoris following ankle joint rotations and 76 ms following knee joint rotations). Second, at least for the ankle joint rotations and the resulting short-latency response observed in the contralateral biceps femoris muscle or nerve stimulation and the resulting cSOL and cGL response, cutaneous afferents do not contribute (Stubbs and Mrachacz-Kersting, 2009; Gervasio et al., 2013). This is despite the fact that the stimulation intensity required to reliably elicit these crossed responses, is 85% of M-max or between 2–3 × motor threshold (Stubbs and Mrachacz-Kersting, 2009; Gervasio et al., 2013, 2015). However, this would also argue against the large diameter Group I afferents as mediators of these crossed responses, since one would expect a facilitation also at lower stimulation intensities where these afferents are first recruited (Pierrot-Deseilligny et al., 1981; Hultborn et al., 1987). Nevertheless, in previous studies where we have blocked the large diameter Group I afferents using ischemia, the crossed response in the cSOL and biceps femoris muscles was significantly depressed or completely absent (Stubbs and Mrachacz-Kersting, 2009; Mrachacz-Kersting et al., 2011). This would indicate that low-threshold afferents mediate the crossed response and indeed evidence suggests that these may also become active at stimulation intensities of 4–5 × motor threshold (Gracies et al., 1994). In a recent publication we also provide evidence that the cGL response size is significantly dependent on the firing rate of secondary spindle afferents arising from muscle spindles located within the cGL (Gervasio et al., 2017). However, since for the cGL we did not test if the response is affected following ischemia, the contribution of other types of afferents cannot be excluded.

When discussing the evidence for the possible afferent contributions to these short-latency-crossed-responses, it is important to also note that the same input such as an electrical stimulation of the iTN, results in a depression of the cSOL and a facilitation of the cGL. Although, the functionality of these plantar flexor muscles during gait is still controversial, some studies indicate that the SOL and GL might work antagonistically (Neptune et al., 2001; Stewart et al., 2007). It is therefore possible that the depression and facilitation observed in the cSOL and cGL muscles respectively, is designed to induce ankle dorsiflexion and knee flexion, with the goal of obtaining a faster swing initiation during walking (Gervasio et al., 2015).

Unlike the short depression observed in the cSOL, the cGL response is comprised of several bursts of facilitation with a longer duration. While the first component, at least in the cGL and the cSOL, is spinally mediated likely through commissural interneurons (Stubbs and Mrachacz-Kersting, 2009; Stubbs et al., 2011; Gervasio et al., 2013), the latter components have latencies compatible with mediation via transcortical reflex loops (Mrachacz-Kersting et al.; Stevenson et al., 2013). Indeed, Stevenson et al. (2013) provided convincing evidence that an unexpectedly imposed knee extension causes a large facilitation in the contralateral biceps femoris muscle at 50% the gait cycle that is mediated via cortical circuits. The results from the current study support that this is also the case for the late burst of the cGL crossed reflex (i.e., the LLC). A cortical contribution is typical for a variety of homonymous and heteronymous reflex responses within the same leg during various tasks (Petersen et al., 1998a; Christensen et al., 2001; Mrachacz-Kersting et al., 2006; Zuur et al., 2009). A cortical contribution also to interlimb reflexes may allow the integration with other sensory information and thus potentially lead to improved adaptation to the circumstances than spinally mediated reflexes (Christensen et al., 2001; Zuur et al., 2009).

It is well known that the MEP of the medial head of the gastrocnemius muscle is modulated during a gait cycle (Schubert et al., 1997), thus this is also likely the case for the GL. One may thus argue that in the current study it would have been necessary to elicit a test MEP so that that the onset of the MEP coincided with the various components of the cGL response. However, as stated in the results section, the entire cGL facilitation has an average duration of 59 ± 27 ms and the facilitation of the MEP or the depression of the background muscle activity following either supra or sub-threshold TMS, was thus quantified in a relatively narrow time window. Due to this and the fact that the experimental procedures were quite time intense, we decided to elicit the test MEP or the suppression alone at only one time point in the gait cycle.

The significant facilitation of the MEP as quantified here, although indicative of a convergence between the MEP and the crossed reflex, does not convey the locality of this convergence. A non-facilitated MEP evoked following transcranial electrical stimulation (TES) would provide more conclusive proof. However, TES is not tolerated by all participants and the induced pain may activate other cortical pathways that likely inhibit cortical neurons. A relatively novel alternative was proposed by van Doornik et al. (2004), TMS applied at intensities below the active motor threshold results in a depression of volitional activity of the target muscle. In line with this, the magnetic stimulation did not elicit any observable MEPs in Experiment 2. Contrarily, a suppression was observed which commenced approximately 10 ms later than the conventional MEP. This depression of the LLC could therefore be due to a neural pathway arising from afferents on the ipsilateral leg, traversing the motor cortex and converging onto cGL motorneurons either directly or via interneurons.

Although the suppression of the LLC when combined with subTMS was seen in all participants in Experiment 2, we never observed a complete suppression in any of the participants. This is in contrast to past studies where a similar technique has been applied to suppress ipsilateral reflex responses (van Doornik et al., 2004; Mrachacz-Kersting et al., 2006). This may reflect additional control mechanisms that contribute to the LLC such as those arising from brain stem structures. In the cat, these have been shown to converge significantly onto the commissural interneurons but also directly onto contralateral motor neurons (Jankowska and Hammar, 2013). Another possibility is that the relatively weak cortical stimulus may recruit insufficient inhibitory circuits within the motor cortex. In Experiment 2 we attempted to adjust the TMS intensity to evoke only a small depression of the ongoing background activation of the cGL (see Figure 5A) to ensure that the target neuron was within the subliminal fringe (Pierrot-Deseilligny and Burke, 2005). It is possible that with a stronger TMS pulse—but still sub-threshold for evoking a MEP—the inhibition would have been larger.

## Conclusion

Interlimb reflexes have a functional role in interlimb coordination (Gervasio et al., 2015). The cortical input to such reflexes enhances their flexibility for a variety of demands such as unexpected perturbations or uneven overground walking surfaces. Further studies are required that address their pattern of convergence onto motoneurons or interneurons which would further enhance the adaptability of the network to task demands. Such convergence has recently been reported between the cSOL inhibition and the Ia inhibitory interneuron that is part of the disynaptic inhibition (Mrachacz-Kersting et al., 2017). However, it may also be possible that the two pathways are converging onto the same motoneuron and thus acting in parallel. In addition, the precise role of the state of the contralateral muscles in shaping the size of the contralateral responses (Gervasio et al., 2017) requires further investigation.

## Author Contributions

NM-K, SG and VM-P conceptualized and designed the study. NM-K and SG collected the data partly with a student group and analyzed the data. NM-K drafted the manuscript. SG completed the statistical analysis. All authors commented on the manuscript and approved the final version.

## Conflict of Interest Statement

The authors declare that the research was conducted in the absence of any commercial or financial relationships that could be construed as a potential conflict of interest.
